# Is preoperative sarcopenia associated with postoperative complications after pelvic exenteration surgery?

**DOI:** 10.1007/s00423-023-02913-5

**Published:** 2023-05-03

**Authors:** Sergei Bedrikovetski, Luke Traeger, Alice A. Jay, Martin K. Oehler, Jonathan Cho, Marcus Wagstaff, Ryash Vather, Tarik Sammour

**Affiliations:** 1 Discipline of Surgery, Faculty of Health and Medical Sciences, School of Medicine, University of Adelaide, Adelaide, South Australia Australia; 2 Colorectal Unit, Department of Surgery, Royal Adelaide Hospital, 5E 332, Port Road, Adelaide, South Australia 5000 Australia; 3 Department of Gynaecological Oncology, Royal Adelaide Hospital, Adelaide, SA 5000 Australia; 4 Centre for Cancer Biology, University of South Australia, Adelaide, South Australia Australia; 5 Discipline of Obstetrics and Gynaecology, Adelaide Medical School, Robinson Research Institute, The University of Adelaide, Adelaide, SA 5005 Australia; 6 Urology Unit, Department of Surgery, Royal Adelaide Hospital, Adelaide, South Australia Australia; 7 Department of Plastic and Reconstructive Surgery, Royal Adelaide Hospital, Adelaide, South Australia Australia

**Keywords:** Sarcopenia, Pelvic exenteration, Nutrition, Postoperative complications

## Abstract

**Purpose:**

Pelvic exenteration (PE) involves radical surgical resection of pelvic organs and is associated with considerable morbidity. Sarcopenia is recognised as a predictor of poor surgical outcomes. This study aimed to determine if preoperative sarcopenia is associated with postoperative complications after PE surgery.

**Methods:**

This retrospective study included patients who underwent PE with an available preoperative CT scan between May 2008 and November 2022 at the Royal Adelaide Hospital and St. Andrews Hospital in South Australia. Total Psoas Area Index (TPAI) was estimated by measuring the cross-sectional area of the psoas muscles at the level of the third lumbar vertebra on abdominal CT, normalised for patient height. Sarcopenia was diagnosed based on gender-specific TPAI cut-off values. Logistic regression analyses were performed to identify risk factors for major postoperative complications with a Clavien-Dindo (CD) grade ≥ 3.

**Results:**

In total, 128 patients who underwent PE were included, 90 of whom formed the non-sarcopenic group (NSG) and 38 the sarcopenic group (SG). Major postoperative complications (CD grade ≥ 3) occurred in 26 (20.3%) patients. There was no detectable association with sarcopenia and an increased risk of major postoperative complications. Preoperative hypoalbuminemia (*P* = 0.01) and a prolonged operative time (*P* = 0.002) were significantly associated with a major postoperative complication on multivariate analysis.

**Conclusion:**

Sarcopenia is not a predictor of major postoperative complications in patients undergoing PE surgery. Further efforts aimed specifically at optimising preoperative nutrition may be warranted.

## Introduction

Pelvic exenteration (PE) is a complex surgical procedure that is considered in patients with advanced disease involving multiple adjacent pelvic organs or compartments [1]. The surgery involves radical en bloc resection of two or more contiguous pelvic organs, followed by reconstruction or diversion of visceral functions and repair of the pelvic defect. It may also include resection of bone or neurovascular contents of the lateral compartment [2]. PE has undergone several modifications since Brunschwig first described it in 1948 to maximise long-term survival and minimise anatomical distortion [3–5]. Nevertheless, PE surgery is technically challenging and traditionally associated with high rates of morbidity and mortality [6, 7]. Therefore, identifying modifiable risk factors for postoperative complications is paramount to improving patient outcomes.

Sarcopenia is progressive generalised depletion of muscle mass, strength, and function, resulting primarily from aging and potentially exacerbated by the effects of cancer, chemotherapy, and malnutrition [8]. The global prevalence of sarcopenia ranges from 10 to 27%; however, future projections predict a dramatic increase in the number of sarcopenic patients in the next 30 years, making sarcopenia a major public health issue [9, 10]. Computed tomography (CT) is routinely performed preoperatively in patients undergoing PE. The cross-sectional view of the psoas muscle at the third lumbar vertebrae (L3) can provide an opportunity to estimate muscle mass and can be used as a surrogate marker for sarcopenia [11]. Two high-quality meta-analyses have shown that sarcopenia has a negative impact on postoperative mortality, major complications, and length of hospital stay in abdominal surgery [12, 13].

More recently, Rees et al. found malnutrition to be a strong risk factor for major complications after PE for rectal cancer. Given that malnutrition is one of the critical pathophysiological causes of sarcopenia, it is plausible that sarcopenia may also be a risk factor for major complications after PE surgery [14]. However, studies investigating the specific relationship between sarcopenia and postoperative complications after PE are scarce due to low patient numbers in a complex patient cohort [15]. This study aims to determine if preoperative sarcopenia is associated with postoperative complications after PE surgery.

## Material and methods

This retrospective cohort study employed the Strengthening the Reporting of Observational Studies in Epidemiology (STROBE) statement [16]. It was approved by the Central Adelaide Local Health Network Human Research Ethics Committee (HREC/17/RAH/470) and informed consent was obtained from all patients. The study was conducted in accordance with the principles of the Declaration of Helsinki.

### Patient selection

Patients who underwent PE for colorectal cancer, gynaecological malignancy, sarcoma, or benign disease between May 2008 and November 2022 at the Royal Adelaide Hospital and St. Andrews Private Hospital in South Australia were retrospectively identified from a prospectively maintained database set up for the PelvEx collaborative as previously reported [17]. All patients were scheduled for PE based on computed tomography (CT), positron emission tomography (PET), or magnetic resonance imaging (MRI), and multi-disciplinary team (MDT) discussion. Total PE was defined as complete en bloc resection of the rectum, genitourinary viscera, and reproductive organs. Anterior PE included resection of the bladder with reproductive organs. Posterior PE included resection of the rectum and reproductive organs, with preservation of the bladder. Modified PE referred to any PE with colonic anastomosis. Infra-levator PE included a wider, extended dissection below the level of the levator muscles and flap reconstruction [18–20]. Patients were included if they had an accessible abdominal CT scan within 3 months prior to surgery or before the commencement of neoadjuvant therapy. Patients were excluded if height was not recorded or if the CT scan was not accessible via Picture Archiving and Communication System (PACS) or InteleViewer™ Australia.

### Data collection

Baseline data included sex, age, body mass index (BMI), American Society of Anaesthesiologists score (ASA), preoperative haemoglobin, total protein, and albumin level, tumour type, primary or recurrent disease, palliative resection, clinical stage, neoadjuvant therapy, time from radiation to resection, and adjuvant therapy. Operative characteristics included operative time, blood loss, intraoperative and postoperative blood transfusion requirement, type of PE surgery, and need for a stoma, bony resection, or flap reconstruction. Pathological and postoperative outcomes were also recorded, including pathological stage based on the American Joint Committee on Cancer (AJCC) Cancer staging manual, resection margin status in curative intent patients (R0 resection was defined as resection margin of > 1 mm, R1 resection was defined as resection margin of < 1 mm, and R2 resection defined as the presence of macroscopic residual disease), length of hospital stay, and 30-day readmission, mortality, and postoperative complications (Clavien-Dindo (CD) and comprehensive complication index (CCI)) [21–23]. Major postoperative complications were defined as CD grade of ≥ 3. Postoperative bleeding was defined as a haemoglobin drop requiring a blood transfusion. Local recurrence was defined as relapse of tumour in the pelvic region. Distant recurrence was defined as relapse of tumour outside the pelvic region (e.g. peritoneal, hepatic, pulmonary, or lymph nodes distant from the resection site). Local and distant recurrence were assessed by clinical investigation and imaging studies. The disease-free survival (DFS) and overall survival (OS) were defined as the interval from start of treatment to the first occurrence of local recurrence, distant recurrence, or death from any cause, respectively).

### Sarcopenia assessment

**Fig. 1:**
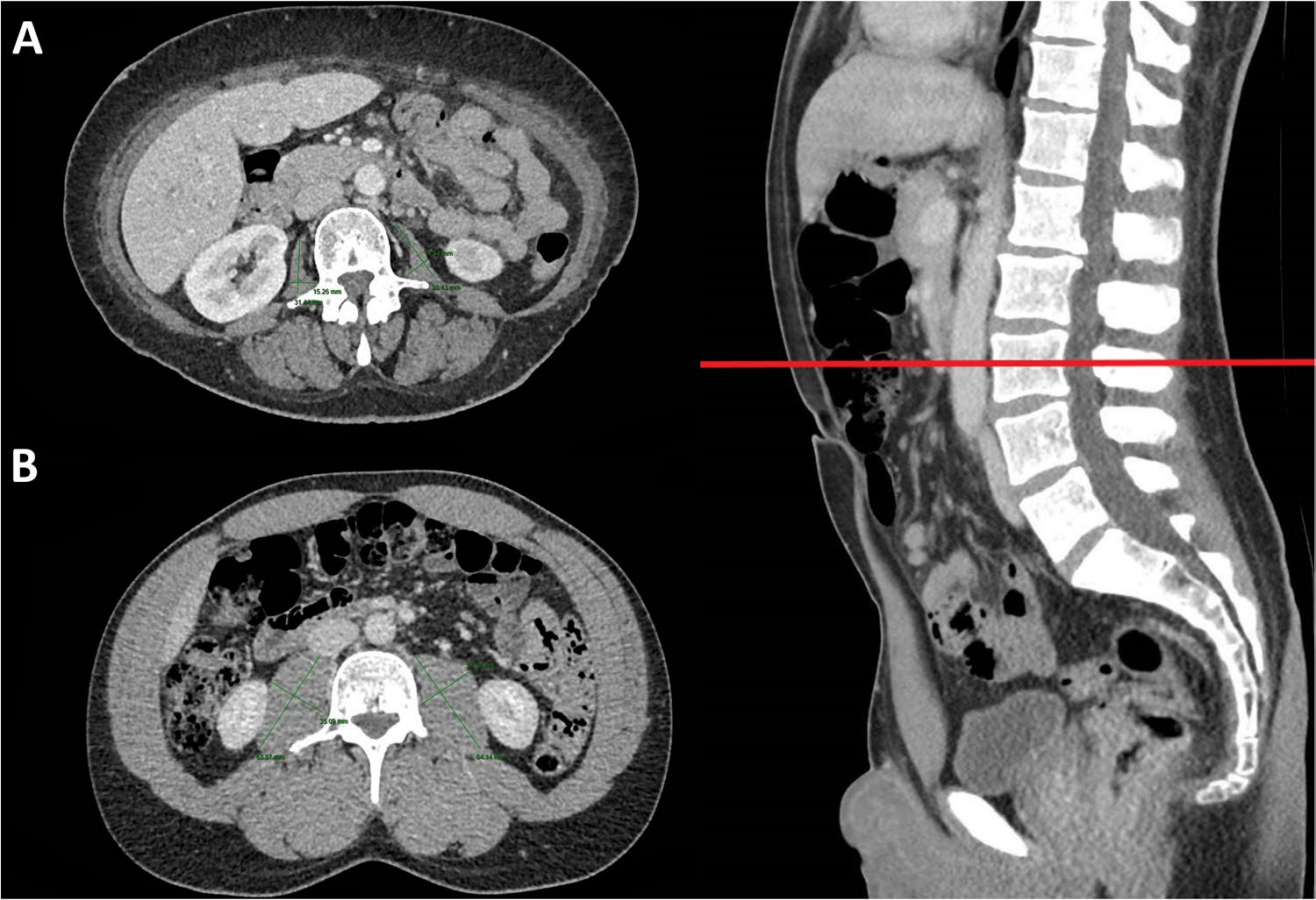
Abdominal computed tomography scans showing the total psoas area measurement in a sarcopenic patient (**A**) and a non-sarcopenic patient (**B**) at the third lumbar vertebra

Preoperative staging CT images were obtained through PACS or InteleViewer™ Australia on standard desktop computer monitors to diagnose pretreatment sarcopenia. Total psoas area (TPA) was assessed in the axial plane from routine abdominal CT at the level of the L3 vertebrae. The cross-sectional area of the left and right psoas muscle was calculated by multiplying the longest anterior to posterior and transverse muscle diameters and added to obtain TPA (Fig. 1) [11]. TPA was then normalised for the patient’s height squared to calculate the Total Psoas Area Index (TPAI). Sarcopenia was defined using previously validated gender-specific cut-off points: < 385 mm^2^/m^2^ in females and < 545 mm^2^/m^2^ in males [24].

### Statistical analysis

The analysis was performed on SPSS for Macintosh (Version 28; IBM Corp., Armonk, NY). Parametricity was determined using the Shapiro–Wilk test. Normally distributed data were expressed as mean ± SD and nonparametric data as median ± range. Univariate analysis was carried out using the *χ*^2^ test for categorical variables, the Mann–Whitney *U* test for nonparametric continuous variables, and a Student *t*-test for parametric continuous variables. Logistic regression analysis was performed to identify risk factors for major postoperative complications. Univariate analyses were performed for each variable and multivariate analysis included variables with a significance level of *P* < 0.05. Sarcopenia was included in the multivariable analysis irrespective of significance level. Odds ratios (OR) were reported with a 95% confidence interval. Results were considered statistically significant if *P* < 0.05. Local recurrence, distant recurrence, DFS, and OS at 3 and 5 years were analysed using the Kaplan–Meier method and compared using a log-rank (Mantel-Cox) test for statistical significance. Patients alive with no or stable disease were censored with the date of last follow-up.

## Results

**Fig. 2:**
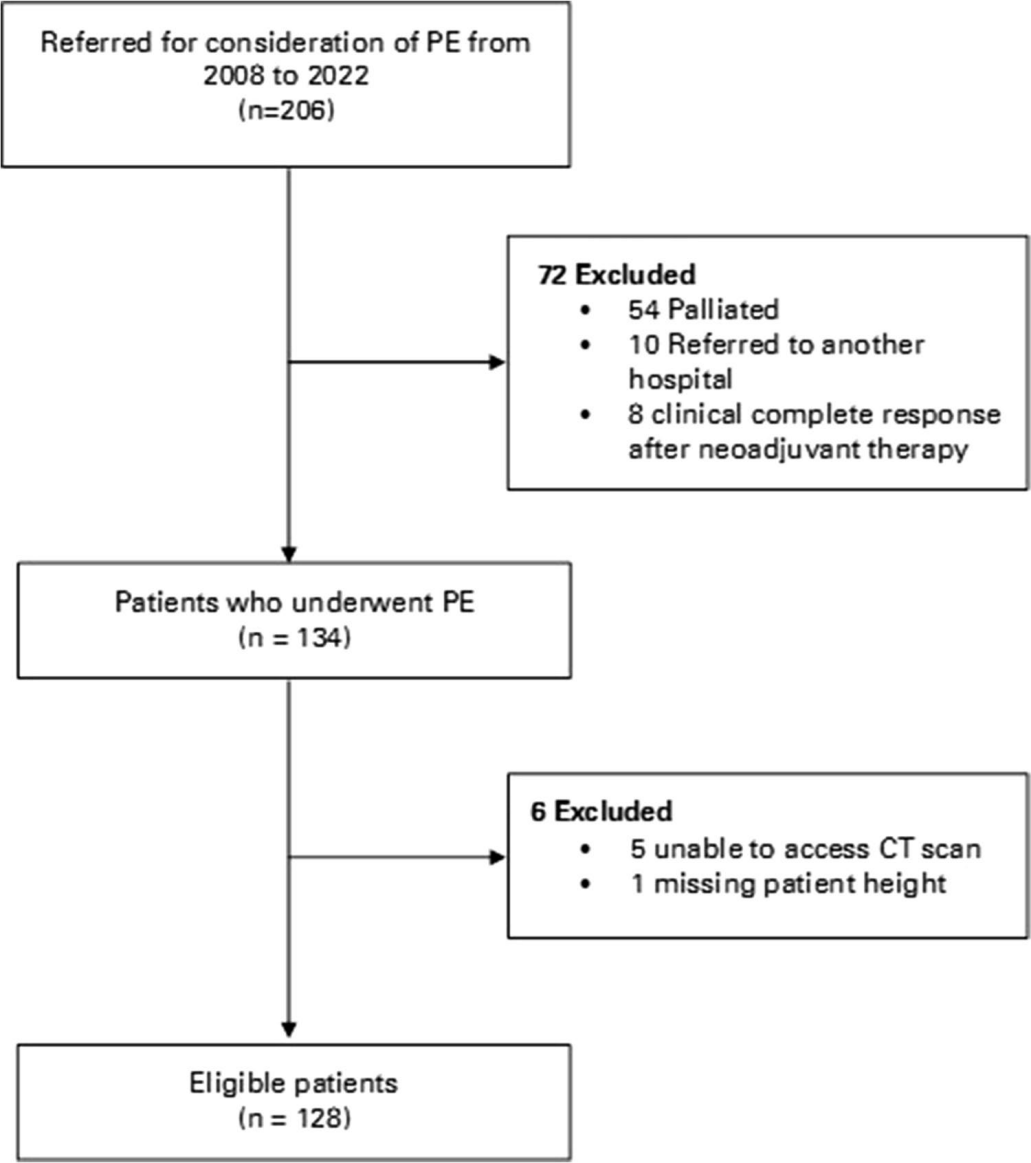
Flowchart depicting the patient selection process

A total of 206 patients were referred for consideration of PE between May 2008 and November 2022. Seventy-two were excluded because they were either for palliative treatment only, referred to another hospital or achieved a clinical complete response after neoadjuvant treatment. Of the 134 patients who underwent PE, five were excluded owing to the inability to access the CT scan and one due to missing the patient’s height. A total of 128 patients were included in the study (Fig. 2).

Baseline characteristics are presented in Table 1. A higher proportion of patients were female (68.8%) with a median (range) age of 64 years (20–90). The median (range) TPAI of all patients was 534mm^2^/m^2^ (121–1352). Based on TPAI calculations, 38 (29.7%) patients were included in the sarcopenic group (SG) and 90 (70.3%) in the non-sarcopenic group (NSG). Patients in the SG were more likely to have a lower median BMI, a higher prevalence of primary cancers, and increased use of neoadjuvant treatment compared to the NSG (*P* < 0.001, *P* = 0.004, *P* = 0.002, respectively). There were no significant differences in the remaining baseline characteristics between the groups.

**Table 1:** Baseline characteristics

Variable	Total (*n* = 128)	Sarcopenic (*n* = 38)	Non-sarcopenic (*n* = 90)	*P*-value
Age (years)	64 (20–90)	63 (27–75)	64 (20–90)	0.28
Gender				0.09
Female	88 (68.8)	22 (57.9)	66 (73.3)	
Male	40 (31.3)	16 (42.1)	24 (26.7)	
BMI (kg/m^2^)	26 (14–67)	23 (14–45)	27 (17–67)	** < 0.001**
TPA	1433 (349–4046)	944 (349–1659)	1641 (1009–4046)	** < 0.001**
TPAI	534 (121–1352)	358 (121–524)	602 (389–1352)	** < 0.001**
ASA				0.88
1–2	72 (56.3)	21 (55.3)	51 (56.7)	
3–4	56 (43.8)	17 (44.7)	39 (43.3)	
Haemoglobin (g/l)				0.47
< 110	32 (25.4)	11 (29.7)	21 (23.6)	
≥ 110	94 (74.6)	26 (70.3)	68 (76.4)	
Total protein (g/l)				0.80
< 60	9 (7.2)	3 (8.1)	6 (6.8)	
≥ 60	116 (92.8)	34 (91.9)	82 (93.2)	
Albumin (g/l)				0.13
< 34	45 (36.0)	17 (45.9)	28 (31.8)	
≥ 34	80 (64.0)	20 (54.1)	60 (68.2)	
Tumour type				0.08
Colorectal	76 (59.4)	25 (67.6)	50 (55.6)	
Gynaecological	38 (29.7)	7 (18.9)	31 (34.4)	
Other cancer (bladder, sarcoma)	4 (3.1)	0 (0.0)	4 (4.4)	
Benign	10 (7.8)	5 (13.2)	5 (5.6)	
Primary or recurrence				**0.004**
Primary	101 (78.9)	36 (94.7)	65 (72.2)	
Recurrence	27 (21.1)	2 (5.3)	25 (27.8)	
Palliative resection	18 (14.1)	7 (18.4)	11 (12.2)	0.36
Clinical T stage				1.00
0	2 (1.9)	0 (0.0)	2 (2.8)	
1	3 (2.9)	1 (3.0)	2 (2.8)	
2	2 (1.9)	0 (0.0)	2 (2.8)	
3	13 (12.5)	4 (12.1)	9 (12.7)	
4a	46 (44.2)	15 (45.5)	31 (43.7)	
4b	38 (36.5)	13 (39.4)	25 (35.2)	
Clinical N stage				0.08
0	59 (56.7)	14 (43.8)	45 (62.5)	
1–2	45 (43.3)	18 (56.3)	27 (37.5)	
Neoadjuvant therapy				**0.002**
None	65 (50.8)	14 (36.8)	51 (56.7)	
CRT	31 (24.2)	9 (23.7)	22 (24.4)	
TNT	20 (15.6)	13 (34.2)	7 (7.8)	
SCRT	6 (4.7)	0 (0.0)	6 (6.7)	
Chemotherapy	5 (3.9)	1 (2.6)	4 (4.4)	
Brachytherapy	1 (0.8)	1 (2.6)	0 (0.0)	
Time from radiotherapy to resection (weeks)	14 (1–300)	16 (10–150)	12 (1–300)	0.18
Adjuvant therapy	59 (48.8)	19 (54.3)	40 (46.5)	0.44

*ASA*, American Society of Anaesthesiologists physical status; *BMI*, body mass index; *TPA*, total psoas area; *TPAI*, Total Psoas Area Index; *CRT*, chemoradiotherapy; *TNT*, total neoadjuvant therapy; *SCRT*, short-course radiotherapy

Total PE and posterior PE were the two most common procedures, accounting for 64.9% of operations (Table 2). Fifty-nine (46.1%) patients underwent a flap procedure, and 12 (9.4%) patients had a bone resection. Median (range) operative time for the whole cohort was 331 min (120–1080). Overall median (range) blood loss and transfusion requirement were 500 ml (50–4500) and 2 units (1–23), respectively. The postoperative bleeding rate was considerably higher in the SG compared to NSG (21.1% vs 3.3%, *P* = 0.003), but there was no significant difference in the transfusion requirement between the groups (*P* = 0.86).

**Table 2:** Operative characteristics

Variable	Total (*n* = 128)	Sarcopenic (*n* = 38)	Non-sarcopenic (*n* = 90)	*P*-value
PE type				0.92
Total	33 (25.8)	11 (28.9)	22 (24.4)	
Posterior	50 (39.1)	15 (39.5)	35 (38.9)	
Anterior	7 (5.5)	1 (2.6)	6 (6.7)	
Modified	15 (11.7)	5 (13.2)	10 (11.1)	
Infralevator	7 (5.5)	1 (2.6)	6 (6.7)	
Other	16 (12.5)	5 (13.2)	11 (12.2)	
Operation time (min)	331 (120–1080)	300 (120–923)	360 (125–1080)	0.83
Blood loss (ml)	500 (50–4500)	500 (100–4500)	500 (50–4000)	0.91
Blood transfusions				0.86
No	76 (59.4)	23 (60.5)	53 (58.9)	
Yes	52 (40.6)	15 (39.5)	37 (41.1)	
pRBCs units transfused	2 (1–23)	3 (1–23)	2 (1–20)	0.18
Flap construction				0.85
No	69 (53.9)	20 (52.6)	49 (54.4)	
Yes	59 (46.1)	18 (47.4)	41 (45.6)	
Bone resection				0.34
No	116 (90.6)	33 (86.8)	83 (92.2)	
Yes	12 (9.4)	5 (13.2)	7 (7.8)	
Stoma				0.23
No	15 (11.7)	2 (5.3)	13 (14.4)	
Yes	113 (88.3)	36 (94.7)	77 (85.6)	

*pRBCs*, packed red blood cells; *PE*, pelvic exenteration; *MIS*, minimally invasive surgery

Pathological and postoperative outcomes are summarised in Table 3. R0 resection was achieved in 89 (89.9%) patients treated with curative intent, whereas 8 (8.1%) patients had a R1 resection and 2 (2.0%) had a R2 resection. However, the difference in margin status was not significantly different between groups (*P* = 0.13). The overall mortality rate within 30 days of surgery was 1.6%. In total, 89 (69.5%) patients developed complications, of whom 63 (70.8%) had minor complications (CD grades 1–2), and 26 (29.2%) had major complications (CD grade ≥ 3). There was no significant difference in CD complications, CCI, length of hospital stay, 30-day readmission, and mortality rates between the two groups.

**Table 3:** Pathological and postoperative outcomes

Variable	Total (*n* = 128)	Sarcopenic (*n* = 38)	Non-sarcopenic (*n* = 90)	*P*-value
Pathological T stage				0.77
0/Tis	7 (6.7)	2 (6.3)	5 (6.9)	
1	5 (4.8)	2 (6.3)	3 (4.2)	
2	12 (11.5)	4 (12.5)	8 (11.1)	
3	24 (23.1)	10 (31.3)	14 (19.4)	
4a	24 (23.1)	6 (18.8)	18 (25.0)	
4b	32 (30.8)	8 (25.0)	24 (33.3)	
Pathological N stage				0.52
0	69 (67.6)	21 (72.4)	48 (65.8)	
1–2	33 (32.4)	8 (27.6)	25 (34.2)	
Pathological M stage				0.11
0	88 (79.3)	23 (69.7)	65 (83.3)	
1	23 (20.7)	10 (30.3)	13 (16.7)	
Margin status for curative intent				0.13
R0	89 (89.9)	25 (96.2)	64 (87.7)	
R1	8 (8.1)	0 (0.0)	8 (11.0)	
R2	2 (2.0)	1 (3.8)	1 (1.4)	
Postoperative complications				
Wound breakdown/necrosis	23 (18.0)	5 (13.2)	18 (20.0)	0.36
Intraabdominal collection	7 (5.5)	2 (5.3)	5 (5.6)	1.00
Anastomotic leak	2 (1.6)	1 (2.6)	1 (1.1)	0.53
Urosepsis	20 (15.6)	6 (15.8)	14 (15.6)	0.97
Urinary leak	7 (5.5)	3 (7.9)	4 (4.4)	0.43
Bleeding	11 (8.6)	8 (21.1)	3 (3.3)	**0.003**
Length of hospital stay (days)	12 (4–93)	15 (4–66)	11 (5–93)	0.59
30-day readmission	16 (12.5)	2 (5.3)	14 (15.6)	0.15
30-day mortality	2 (1.6)	0 (0.0)	2 (2.2)	1.00
Clavien-Dindo grade of complications				0.08
Minor (1–2)	63 (70.8)	24 (82.8)	39 (65.0)	
Major (≥ 3)	26 (29.2)	5 (17.2)	21 (35.0)	
CCI	22.7 (0–100)	22.7 (0–49.5)	22.7 (0–100)	0.86
CCI excluding patients with no complications	29.6 (9–100)	29.6 (21–50)	30.2 (9–100)	0.25

*CCI*, comprehensive complication index

The results of the univariate logistic regression analysis are shown in Table 4. Low preoperative albumin of < 34 g/l and a longer operative time of ≥ 330 min were found to be significantly associated with major postoperative complications. In the multivariate logistic regression analysis, preoperative hypoalbuminemia (OR: 3.52, 95%CI: 1.33–9.31, *P* = 0.01), and a longer operative time (OR: 5.83, 95%CI: 1.93–17.61, *P* = 0.002) retained their significant association with major postoperative complications. In the univariate and multivariate analyses, sarcopenia was not predictive of major postoperative complications (Table 5).

**Table 4:** Univariate logistic regression analysis predicting a major postoperative complication (Clavien-Dindo grade ≥ 3)

Variable	*N* (%) with a major postoperative complication	OR (95%CI)	*P*-value
Sex			
Female	19 (21.6)	1.30 (0.50–	0.60
Male	7 (17.5)	3.39)	
Age (years)			
< 70	17 (20.0)	1.06 (0.42–	0.90
≥ 70	9 (20.9)	2.62)	
BMI (kg/m^2^)			
< 25	8 (18.2)	1.33 (0.52–	0.55
> 25	18 (22.8)	3.36)	
Haemoglobin (g/l)			
< 110 ≥ 110	10 (31.3) 16 (17.0)	2.22 (0.88–5.57)	0.09
Total protein (g/l)			
< 60	4 (44.4)	3.42 (0.85–	0.08
≥ 60	22 (19.0)	13.78)	
Albumin (g/l)			
< 34	15 (33.3)	3.14 (1.29–	**0.012**
≥ 34	11 (13.8)	7.62)	
ASA			
1–2	11 (15.3)	2.03 (0.85–	0.11
3–4	15 (26.8)	4.86)	
Sarcopenia			
No	21 (23.3)	0.50 (0.17–	0.20
Yes	5 (13.2)	1.44)	
Tumour type			
Colorectal	14 (18.4)	Reference	
Gynaecological	9 (23.7)	1.37 (0.53–3.54)	0.51
Other	3 (21.4)	1.21 (0.30–4.91)	0.79
Primary or recurrence			
Primary	17 (16.8)	2.47 (0.95–6.42)	0.06
Recurrence	9 (33.3)		
Palliative resection			
No	23 (20.9)	0.76 (0.20–	0.68
Yes	3 (16.7)	2.84)	
Clinical T stage			
0–3	5 (25.0)	0.82 (0.26–	0.73
4	18 (21.4)	2.55)	
Clinical N stage			
N0	15 (25.4)	0.73 (0.29–	0.52
N +	9 (20.0)	1.87)	
Neoadjuvant therapy			
No	13 (20.0)	1.04 (0.44–	0.93
Yes	13 (20.6)	2.46)	
Time from radiotherapy to resection			
≥ 14 weeks	5 (17.2)	0.63 (0.17–2.27)	0.48
< 14 weeks	7 (25.0)		
Adjuvant therapy			
No	13 (21.0)	0.96 (0.40–	0.93
Yes	12 (20.3)	2.32)	
Operation time, minutes			
≥ 330	20 (32.3)	5.14 (1.78–14.84)	**0.002**
< 330	5 (8.5)		
Blood loss (ml)			
≥ 500	15 (22.4)	1.11 (0.41–3.06)	0.84
< 500	7 (20.6)		
Flap construction			
No	13 (18.8)	1.22 (0.51–2.88)	0.66
Yes	13 (22.0)		
Bone resection			
No	23 (19.8)	1.35 (0.34–5.38)	0.67
Yes	3 (25.0)		

*OR*, odds ratio; *CI*, confidence interval; *ASA*, American Society of Anaesthesiologists physical status; *BMI*, body mass index; *pRBCs*, packed red blood cells

**Table 5:** Multivariate logistic regression analysis predicting a major postoperative complication (Clavien-Dindo grade ≥ 3)

Variable	OR	95%CI	*P*-value
Sarcopenia	0.47	0.15–1.53	**0.211**
Albumin < 34	3.92	1.44–10.69	**0.008**
Operation time ≥ 330	5.59	1.83–17.08	**0.003**

*OR*, odds ratio; *CI*, confidence interval

**Fig. 3:**
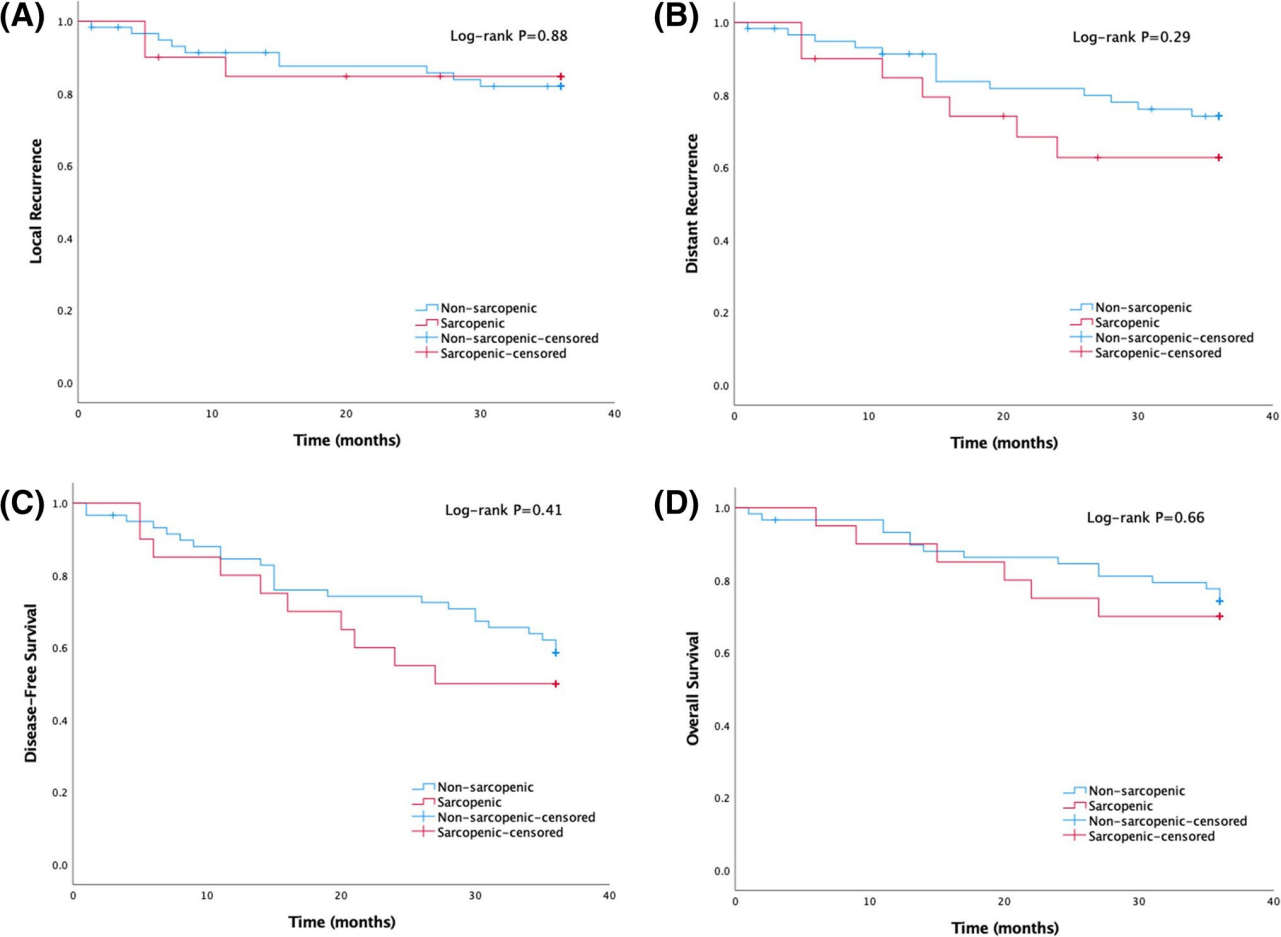
Kaplan–Meier estimates of 3-year (**A**) local recurrence, (**B**) distant recurrence, (**C**) disease-free survival, and (**D**) overall survival in patients treated with pelvic exenteration stratified according to sarcopenic status

**Fig. 4:**
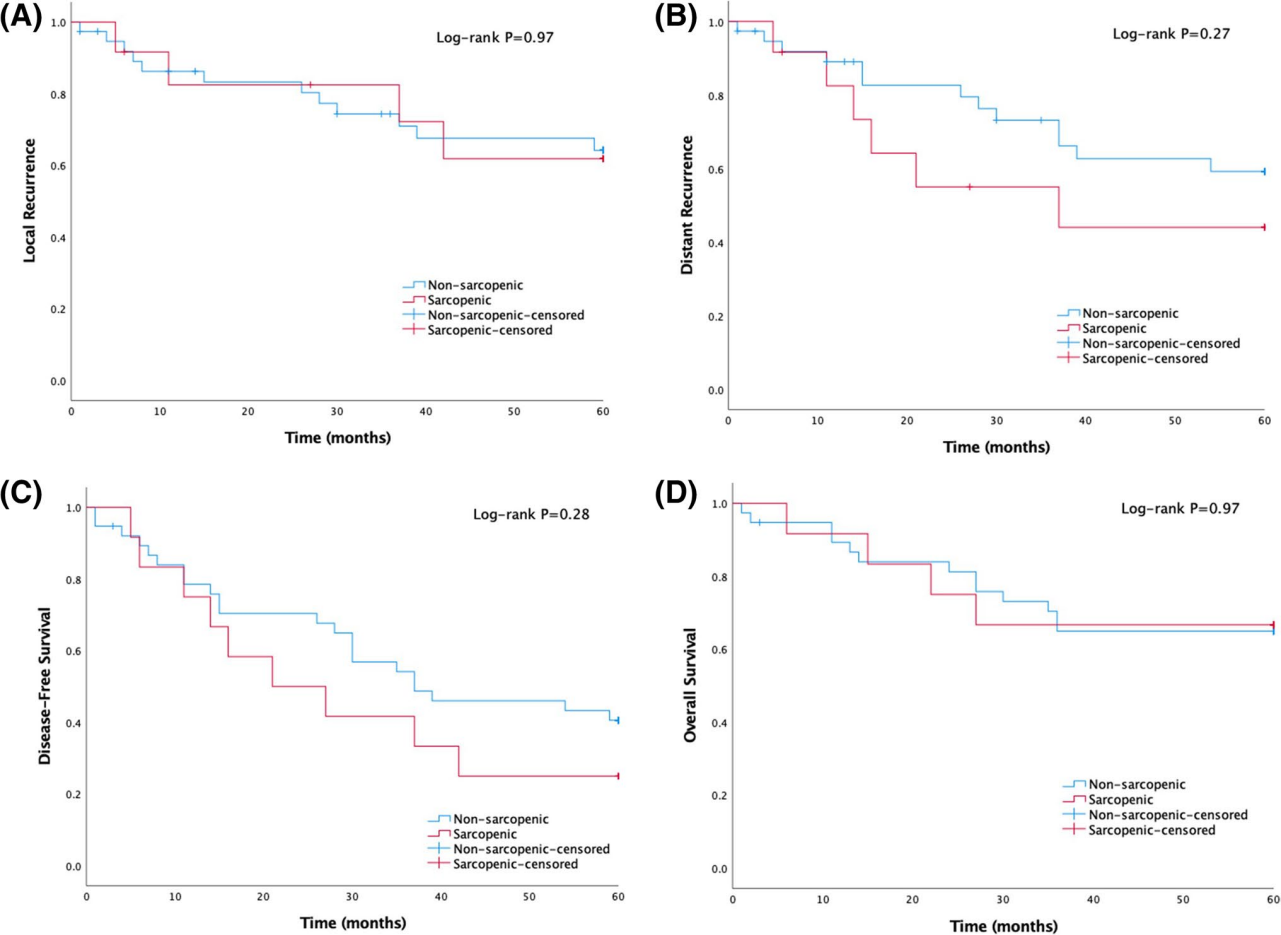
Kaplan–Meier estimates of 5-year (**A**) local recurrence, (**B**) distant recurrence, (**C**) disease-free survival, and (**D**) overall survival in patients treated with pelvic exenteration stratified according to sarcopenia status

Kaplan–Meier curves depicting the association of sarcopenic status with local recurrence, distant recurrence, DFS, and OS are presented in Figs. 3 and 4. Median length of follow-up was 45.1 months (interquartile range, 30.6–73.0). The 3-year local recurrence, distant recurrence, DFS, and OS rates were 16.5%, 26.6%, 43.0%, and 26.6%, respectively. The 5-year local recurrence, distant recurrence, DFS, and OS rates were 32.0%, 38.0%, 62.0%, and 34.0%, respectively. There was no statistically significant difference in 3-year local recurrence, distant recurrence, DFS, and OS between sarcopenic and non-sarcopenic patients who underwent PE (*P* = 0.88, *P* = 0.29, *P* = 0.41, *P* = 0.66, respectively). Neither was it possible to detect a significance in 5-year local recurrence, distant recurrence, DFS, and OS between those who were sarcopenic and those who were non-sarcopenic (*P* = 0.97, *P* = 0.27, *P* = 0.28, *P* = 0.97, respectively).

## Discussion

This study has shown that preoperative sarcopenia is not associated with major postoperative complications in patients undergoing PE surgery. However, on multivariate analysis, patients with preoperative hypoalbuminemia and longer operative time were found to have an increased risk of developing major postoperative complications. Moreover, our results demonstrate that sarcopenic status does not affect 3-year or 5-year local recurrence, distant recurrence, DFS, and OS rates in patients undergoing PE surgery.

The findings in this study are consistent with the conclusions of a smaller series of 64 patients, which evaluated the impact of sarcopenia on postoperative complications after PE surgery [15]. They concluded that preoperative sarcopenia measured radiologically was not sensitive enough to predict postoperative complications and outcomes in oncologic patients following PE surgery. Furthermore, Rees et al. investigated the impact of body composition on postoperative outcomes after PE surgery in 227 patients with locally advanced and locally recurrent rectal cancer [25]. Of the body composition variables investigated, patients with low skeletal muscle mass, muscle wasting, and low skeletal muscle density (SMD) were not associated with major postoperative complications (CD grade ≥ 3). This finding is in line with a smaller series showing sarcopenia was not predictive of major postoperative complications in 32 patients with recurrent gynaecological malignancy who underwent PE surgery [26].

The above findings suggest that sarcopenia is not predictive of major postoperative complications after PE surgery. This contradicts evidence from other types of abdominal surgery.^12,13^ It may be that higher rate of overall complications associated with the surgical complexity of PE as an operation (compared with less extensive abdominal operations) confounds any sarcopenia-related factors. Consistent with this, we noted an association between operative time and postoperative complications which is likely representative of the more complex surgical resections predisposing patients to major complications [27]. A large study from the PelvEx collaborative similarly identified prolonged operative time as negatively associated with 30-day complications [28].

The present study also demonstrates that sarcopenic patients are at greater risk of postoperative bleeding compared to non-sarcopenic patients after PE surgery. The exact aetiology for increased postoperative bleeding in sarcopenia remains elusive. However, sarcopenia has been previously linked to perioperative blood transfusion requirements in patients undergoing rectal cancer surgery [29]. Chen et al. used machine learning to identify predictors of postoperative bleeding for patients undergoing colorectal surgery [30]. They showed decreased mobility, lower activity level, and poor nutrition to be among the most important predictors of postoperative bleeding. Interestingly, all three of the above risk factors contribute to the development of sarcopenia, which we speculate may account for the increased postoperative bleeding rate observed in our study. These findings support the idea that a patient’s functional condition is a key factor for adverse outcomes after PE surgery [31]. It is important to note that diagnosing sarcopenia via TPAI alone rather than including strength and physical performance components impedes determination of sarcopenic severity [8]. A refined method of diagnosing sarcopenia using physical performance could allow for targeted interventions to those with a more severe sarcopenia, resulting in improved patient outcomes.

Despite not identifying a significant difference in postoperative complications between the SG and NSG, we did identify preoperative hypoalbuminemia as an independent risk factor for major postoperative complications following PE surgery. Similar to our findings, recent Danish and USA studies both found hypoalbuminemia to be independently associated with major postoperative complications after PE [32, 33]. Malnutrition has been shown to adversely affect postoperative outcomes after major abdominal surgery [34, 35]. Moreover, the incidence of malnutrition preoperatively ranges between 24 and 33% and postoperatively around 51–53% in patients undergoing PE surgery [36, 37]. The majority of patients in the present study had advanced malignancy. These patients are at high risk of malnutrition and subsequent hypoalbuminemia due to cancer cachexia, increased metabolism, poor dietary intake, and the effect of tumour necrosis factor-alpha altering liver protein synthesis.^35^ Although not well established, targeted interventions such as preoperative nutritional support as part of a prehabilitation programme may improve outcomes after PE surgery and require further investigation. In addition, utilising indirect calorimetry (where feasible) to measure energy expenditure more accurately may better inform nutrition targets and guide nutrition prescriptions at opportunistic timepoints in PE patients’ treatment and surgical journey, with the goal of optimising patient outcomes.

Current research suggests that loss of skeletal muscle mass has been associated with DFS but not OS in patients with locally advanced rectal cancer undergoing neoadjuvant chemoradiotherapy [38]. We found that patients undergoing PE who were sarcopenic did not diverge significantly from those who were non-sarcopenic in local recurrence, distant recurrence, DFS, and OS rates. In coherence with our results, Rees et al. showed body composition variables had no effect on overall survival in their multivariable analysis [25]. Interestingly, a small Austrian study reported that preoperative low muscle attenuation, an established factor for muscle depletion, was associated with shorter survival in patients with recurrent gynaecological malignancies treated with PE [26]. However, skeletal muscle index remains a key measurement to diagnose sarcopenia and neither study were able to show an association between skeletal muscle index and survival in their respective populations, further confirming our results [8].

The main limitations of our study are its retrospective design and relatively small sample size. However, given the scarcity of patients who undergo PE surgery, it is challenging to perform a robust sarcopenia analysis in a larger and more homogenous patient population. Furthermore, evaluating a population treated in a tertiary care centre could increase the likelihood of referral bias by including patients with a higher surgical risk. It is possible that because the overall complication rate when prospectively collected is so high in these patients (independent of patient fitness), preoperative predictive variables do not have enough of a detectable independent impact compared with the extent of surgery itself. Analysis and correlation with specific individual postoperative complications (as opposed to overall complications) was thought to be out of the scope of the current analysis. Lastly, low muscle mass based on radiological imaging may be suggestive yet not diagnostic of sarcopenia unless confirmed by assessing muscle strength or physical performance.

## Conclusions

Sarcopenia was not associated with major postoperative complications or a survival detriment in patients undergoing PE surgery. However, other perioperative risk factors such as preoperative hypoalbuminemia predict major postoperative complications. Further research is required to determine whether these perioperative risk factors can be modified and will diminish major complication rates in patients undergoing PE.

## Data Availability

The data that support the findings of this study are available on request from the corresponding author (SB). The data are not publicly available due to their containing information that could compromise the privacy of research participants.

## References

[1] Pawlik TM, Skibber JM, Rodriguez-Bigas MA (2006). Pelvic exenteration for advanced pelvic malignancies. Ann Surg Oncol.

[2] Austin KK, Herd AJ, Solomon MJ, Ly K, Lee PJ (2016). Outcomes of pelvic exenteration with en bloc partial or complete pubic bone excision for locally advanced primary or recurrent pelvic cancer. Dis Colon Rectum.

[3] Brunschwig A (1948). Complete excision of pelvic viscera for advanced carcinoma; a one-stage abdominoperineal operation with end colostomy and bilateral ureteral implantation into the colon above the colostomy. Cancer.

[4] PelvExCollaborative (2019). Pelvic exenteration for advanced nonrectal pelvic malignancy. Ann Surg..

[5] Brown KGM, Solomon MJ, Koh CE (2017). Pelvic exenteration surgery: the evolution of radical surgical techniques for advanced and recurrent pelvic malignancy. Dis Colon Rectum.

[6] PelvEx C (2019). Surgical and survival outcomes following pelvic exenteration for locally advanced primary rectal cancer: results from an international collaboration. Ann Surg.

[7] Nielsen MB, Rasmussen PC, Lindegaard JC, Laurberg S (2012). A 10-year experience of total pelvic exenteration for primary advanced and locally recurrent rectal cancer based on a prospective database. Colorectal Dis.

[8] Cruz-Jentoft AJ, Bahat G, Bauer J, Boirie Y, Bruyere O, Cederholm T (2019). Sarcopenia: revised European consensus on definition and diagnosis. Age Ageing.

[9] Petermann-Rocha F, Balntzi V, Gray SR, Lara J, Ho FK, Pell JP (2022). Global prevalence of sarcopenia and severe sarcopenia: a systematic review and meta-analysis. J Cachexia Sarcopenia Muscle.

[10] Ethgen O, Beaudart C, Buckinx F, Bruyere O, Reginster JY (2017). The future prevalence of sarcopenia in Europe: a claim for public health action. Calcif Tissue Int.

[11] Jones KI, Doleman B, Scott S, Lund JN, Williams JP (2015). Simple psoas cross-sectional area measurement is a quick and easy method to assess sarcopenia and predicts major surgical complications. Colorectal Dis.

[12] Hajibandeh S, Hajibandeh S, Jarvis R, Bhogal T, Dalmia S (2019). Meta-analysis of the effect of sarcopenia in predicting postoperative mortality in emergency and elective abdominal surgery. Surgeon.

[13] Jones K, Gordon-Weeks A, Coleman C, Silva M (2017). Radiologically determined sarcopenia predicts morbidity and mortality following abdominal surgery: a systematic review and meta-analysis. World J Surg.

[14] Sieber CC (2019). Malnutrition and sarcopenia. Aging Clin Exp Res.

[15] Hogan S, Rangan A, Ritorni F, Solomon M, Carey S, Lai S (2020). Is preoperative sarcopenia a good predictor of postoperative complications and outcomes after pelvic exenteration surgery?. J Surg Oncol.

[16] Ev Elm, Altman DG, Egger M, Pocock SJ, Gøtzsche PC, Vandenbroucke JP (2007). Strengthening the reporting of observational studies in epidemiology (STROBE) statement: guidelines for reporting observational studies. BMJ..

[17] Traeger L, Bedrikovetski S, Oehler MK, Cho J, Wagstaff M, Harbison J (2022). Short-term outcomes following development of a dedicated pelvic exenteration service in a tertiary centre. ANZ J Surg.

[18] PelvEx C (2018). Factors affecting outcomes following pelvic exenteration for locally recurrent rectal cancer. Br J Surg.

[19] Collaborative TP (2019). Surgical and survival outcomes following pelvic exenteration for locally advanced primary rectal cancer: results from an international collaboration. Ann Surg.

[20] PelvEx C (2019). Changing outcomes following pelvic exenteration for locally advanced and recurrent rectal cancer. BJS Open.

[21] Dindo D, Demartines N, Clavien PA (2004). Classification of surgical complications: a new proposal with evaluation in a cohort of 6336 patients and results of a survey. Ann Surg.

[22] Slankamenac K, Graf R, Barkun J, Puhan MA, Clavien PA (2013). The comprehensive complication index: a novel continuous scale to measure surgical morbidity. Ann Surg..

[23] Amin MB, Greene FL, Edge SB, Compton CC, Gershenwald JE, Brookland RK, Meyer L, Gress DM, Byrd DR, Winchester DP (2017) The eighth edition AJCC cancer staging manual: continuing to build a bridge from a population-based to a more “personalized” approach to cancer staging. CA Cancer J Clin 67(2):93–99. 10.3322/caac.21388. 10.3322/caac.21388 28094848

[24] Fearon K, Strasser F, Anker SD, Bosaeus I, Bruera E, Fainsinger RL (2011). Definition and classification of cancer cachexia: an international consensus. Lancet Oncol.

[25] van Rees JM, Visser E, van Vugt JLA, Rothbarth J, Verhoef C, van Verschuer VMT (2021). Impact of nutritional status and body composition on postoperative outcomes after pelvic exenteration for locally advanced and locally recurrent rectal cancer. BJS Open..

[26] Seebacher V, Rockall A, Nobbenhuis M, Sohaib SA, Knogler T, Alvarez RM (2022). The impact of nutritional risk factors and sarcopenia on survival in patients treated with pelvic exenteration for recurrent gynaecological malignancy: a retrospective cohort study. Arch Gynecol Obstet.

[27] Petruzziello A, Kondo W, Hatschback SB, Guerreiro JA, Filho FP, Vendrame C (2014). Surgical results of pelvic exenteration in the treatment of gynecologic cancer. World J Surg Oncol.

[28] Dudurych I, Kelly ME, Aalbers AGJ, Aziz NA, Abecasis N, Abraham-Nordling M (2020). Predicting outcomes of pelvic exenteration using machine learning. Colorectal Dis.

[29] Jochum SB, Kistner M, Wood EH, Hoscheit M, Nowak L, Poirier J (2019). Is sarcopenia a better predictor of complications than body mass index? Sarcopenia and surgical outcomes in patients with rectal cancer. Colorectal Dis.

[30] Chen D, Afzal N, Sohn S, Habermann EB, Naessens JM, Larson DW (2018). Postoperative bleeding risk prediction for patients undergoing colorectal surgery. Surgery.

[31] Steffens D, Young JM, Solomon M, Beckenkamp PR, Koh C, Vuong K (2019). Preliminary evidence for physical activity following pelvic exenteration: a pilot longitudinal cohort study. BMC Cancer.

[32] Pleth Nielsen CK, Sorensen MM, Christensen HK, Funder JA (2022). Complications and survival after total pelvic exenteration. Eur J Surg Oncol..

[33] Lyell NJ, Kitano M, Smith B, Gleisner AL, Backes FJ, Cheng G (2019). The effect of preoperative nutritional status on postoperative complications and overall survival in patients undergoing pelvic exenteration: a multi-disciplinary, multi-institutional cohort study. Am J Surg.

[34] Gibbs J, Cull W, Henderson W, Daley J, Hur K, Khuri SF (1999). Preoperative serum albumin level as a predictor of operative mortality and morbidity: results from the National VA Surgical Risk Study. Arch Surg.

[35] Moghadamyeghaneh Z, Hwang G, Hanna MH, Phelan MJ, Carmichael JC, Mills SD (2015). Even modest hypoalbuminemia affects outcomes of colorectal surgery patients. Am J Surg.

[36] Beaton J, Carey S, Solomon M, Young J (2013). Preoperative and postoperative nutritional status of patients following pelvic exenteration surgery for rectal cancer. e-SPEN J.

[37] Hogan S, Steffens D, Vuong K, Rangan A, Solomon M, Carey S (2022). Preoperative nutritional status impacts clinical outcome and hospital length of stay in pelvic exenteration patients - a retrospective study. Nutr Health.

[38] Levolger S, van Vledder MG, Alberda WJ, Verhoef C, de Bruin RWF, Uzermans JNM (2018). Muscle wasting and survival following pre-operative chemoradiotherapy for locally advanced rectal carcinoma. Clin Nutr.

